# Crocetin Prevents RPE Cells from Oxidative Stress through Protection of Cellular Metabolic Function and Activation of ERK1/2

**DOI:** 10.3390/ijms21082949

**Published:** 2020-04-22

**Authors:** Padideh Karimi, Ali Gheisari, Sylvia J Gasparini, Hossein Baharvand, Faezeh Shekari, Leila Satarian, Marius Ader

**Affiliations:** 1 CRTD/Center for Regenerative Therapies Dresden, Center for Molecular and Cellular Bioengineering, Technische Universität Dresden, 01307 Dresden, Germany; padideh.karimi_sejzei@mailbox.tu-dresden.de (P.K.); sylvia.gasparini@tu-dresden.de (S.J.G.); 2 CMCB/Center for Molecular and Cellular Bioengineering, Technische Universität Dresden, 01307 Dresden, Germany; ali.gheisari@tu-dresden.de; 3 Department of Stem Cells and Developmental Biology, Cell Science Research Center, Royan Institute for Stem Cell Biology and Technology, ACECR, Tehran 1665659911, Iran; baharvand@royaninstitute.org (H.B.); faezehshekari@gmail.com (F.S.); 4 Department of Developmental Biology, University of Science and Culture, Tehran 1665659911, Iran; 5 Department of Brain and Cognitive Sciences, Cell Science Research Center, Royan Institute for Stem Cell Biology and Technology, ACECR, Tehran 1665659911, Iran

**Keywords:** age-related macular degeneration (AMD), oxidative stress, retinal pigment epithelium (RPE) cells, crocetin, energy production, ERK1/2

## Abstract

Age-related macular degeneration (AMD) is a leading cause for visual impairment in aging populations with limited established therapeutic interventions available. Oxidative stress plays an essential role in the pathogenesis of AMD, damaging the retinal pigment epithelium (RPE), which is essential for the function and maintenance of the light-sensing photoreceptors. This study aimed to evaluate the effects of crocetin, one of the main components of Saffron, on an in vitro RPE model of tert-butyl hydroperoxide (TBHP) induced oxidative stress using ARPE19 cells. The effects of crocetin were assessed using lactate de-hydrogenase (LDH) and ATP assays, as well as immunocytochemistry for cell morphology, junctional integrity, and nuclear morphology. The mechanism of crocetin action was determined via assessment of energy production pathways, including mitochondrial respiration and glycolysis in real-time as well as investigation of extracellular signal-regulated kinase 1/2 (ERK1/2) activation and distribution. Our results show that crocetin pre-treatment protects ARPE19 cells from TBHP-induced LDH release, intracellular ATP depletion, nuclear condensation, and disturbance of junctional integrity and cytoskeleton. The protective effect of crocetin is mediated via the preservation of energy production pathways and activation of ERK1/2 in the first minutes of TBHP exposure to potentiate survival pathways. The combined data suggest that a natural antioxidant, such as crocetin, represents a promising candidate to prevent oxidative stress in RPE cells and might halt or delay disease progression in AMD.

## 1. Introduction

Age-related macular degeneration (AMD), a neurodegenerative disease of the macula region in the retina causing vision impairment and blindness, represents a major economic and social burden worldwide [1,2]. AMD can be classified into two stages: early and late. Early AMD characteristics include drusen formation and pigmentary changes. Later stages are characterized by death and loss of retinal pigment epithelia (RPE) cells and cone photoreceptors and is further sub-divided by two main phenotypes: geographic atrophy (GA), also known as “dry” AMD, and choroidal neovascularization, also known as “wet” AMD, that additionally displays blood vessel ingrowth into the retina from the underlying choroid [3,4]. The incidence of early and late AMD in Europe is currently estimated to be between 14.9 to 21.5 and 3.9 to 4.8 million, respectively [5]. The projected worldwide incidence is expected to rise to 196 million by 2020 and 288 million by 2040 [6]. At present, no effective treatment has been clinically established for dry AMD (i.e., early stage and GA of late stage), whereas for wet AMD (choroidal neovascularization in late stage) approaches to inhibit neovascularization via multiple injections of VEGF blockers or laser photocoagulation have been successfully introduced to the clinic [7,8].

Despite an unknown etiology, AMD is believed to be a complex disease caused by an interaction of both genetic and environmental factors [9,10]. Oxidative stress and inflammation are thought to be the most important environmental factors which play a significant role in the pathogenesis of AMD [11,12]. Oxidative stress refers to the excessive accumulation of reactive oxygen species (ROS) inside the cell [13]. Normally, ROS levels are in balance with the antioxidant defense systems [14], but when this balance is disturbed, intracellular ROS increases and induces peroxidation of membrane lipids, release of aldehydes and damage of proteins and DNA [15,16].

The degeneration of RPE cells is one of the early clinical hallmarks of AMD [17,18]. RPE cells are polarized and specialized epithelial cells that form a monolayer between the neural retina and Bruch’s membrane/choroid forming the blood–retinal barrier. RPE cells are essential for the health and function of photoreceptors by phagocytosing their outer segments, transporting nutrients from the choroid to photoreceptors, and metabolic waste products from photoreceptors through Bruch’s membrane to the choroid [19,20]. These cells also play critical roles in recycling retinoids, absorbing excess light, and maintaining the extracellular matrix [21,22]. To generate sufficient amounts of ATP to perform the afore-mentioned physiological functions, RPE cells have an elevated number of mitochondria and high metabolic activity [23]. In general, mitochondria are the main intracellular source of ROS generation and clearance. All of these characteristics and functions make RPE cells a vulnerable environment for the generation of ROS and increase their susceptibility to oxidative injury [11,24,25,26]. RPE cells are equipped with multiple physiological antioxidant defense mechanisms, however, chronic and long term exposure to oxidative stress during aging causes accumulation of ROS and mitochondrial damage due to a reduction in antioxidative capacity [14,27,28,29].

Regarding the diminishment of RPE cell antioxidative capacity in aging, upholding the antioxidant defense systems with specific dietary and supplementary antioxidants represents a potential prevention or therapy for macular degeneration [30]. In support of this idea, a number of studies confirm that some natural antioxidant compounds can act as ROS scavengers [28,31], enhance antioxidant enzymes [32], and induce or inhibit signaling pathways and gene expression related to stress response, cell death, and survival pathways [19,33,34,35]. Besides that, other approaches have been explored to preserve RPE cells from oxidative stress, such as sigma receptor ligands [36].

Saffron, a well-known spice, is noteworthy for its long history of medical benefits [37]. The therapeutic potential of saffron in ocular diseases has been shown in experimental, animal, and human studies [38,39,40,41]. In the case of AMD, its potential to rescue visual impairment has been reported in clinical trials [42,43,44,45,46,47], however, it is still under discussion which component(s) of saffron is responsible for its beneficial effects. Crocin and crocetin are the main active components of saffron. The positive effects of crocin have been shown in an in vitro model of AMD induced by hydrogen peroxide (H_2_O_2_) treatment [48]. Interestingly, orally administrated crocin is hydrolyzed to crocetin during digestion [49], therefore, it is hypothesized that the retino-protective influence of ingested saffron is caused by crocetin which reaches the RPE/retina following intestinal absorption [50]. Crocetin (8,8-diapo-8,8-carotenoic acid) with elementary composition of C_20_H_24_O_4_ and molecular weight of 328.4, is the aglycon of crocin. Its structure consists of seven double bonds and four methyl groups besides two carboxyl groups which stabilize the polyene chain at the terminals (Supplementary Figure S2A) [51]. Crocetin possesses multiple pharmacological activities, including anti-oxidative [52,53], anti-inflammatory [54], anti-cancer [55], and cardio-protective [56] effects. Furthermore, its positive effects have been investigated in animal models of retinal damage [50,57,58], yet, its effect in AMD, and particularly damaged RPE, has not been studied.

The aim of this study is to investigate the direct effect of crocetin in preventing RPE cell damage, as it is probable that RPE deterioration is an initiator of AMD development. Therefore, an oxidative stress model of RPE cells was established by incubating ARPE19 cells with tert-butyl hydroperoxide (TBHP). TBHP is metabolized to non-radical and free radical products via two-electron reduction and one-electron reduction/oxidation [59]. Afterward, TBHP-derived products attack biomolecules and cause lipid peroxidation, GSH depletion, permeabilization of cellular and mitochondrial membrane, and impaired ATP synthesis [29,59,60,61]. Here we show that pretreatment of TBHP-stressed ARPE19 cells with crocetin positively influenced metabolic functions, including mitochondrial respiration and glycolytic instigation, as well as membrane integrity via activation of the extracellular signal-regulated kinase (ERK) pathway, a member of the mitogen-activated protein kinase (MAPK) signaling cascade. Furthermore, the crocetin-induced protection is comparable with well-known antioxidants, such as vitamin C or vitamin E.

## 2. Results

### 2.1. Establishment of An In Vitro Oxidative Stress Model Using Exposure of ARPE19 Cells to TBHP

The ARPE19 cell line recapitulates structural, morphological and functional features of RPE cells [62] and was used to design an in vitro model of oxidative stress. As ARPE19 cells with longer culture periods (>1 week) have been reported to be less vulnerable to oxidative stress [63], all experiments were performed two weeks after seeding in 96-well plates with the aim to establish a robust oxidative stress model leading to significant cell damage and cell death [64,65].

**Figure 1:**
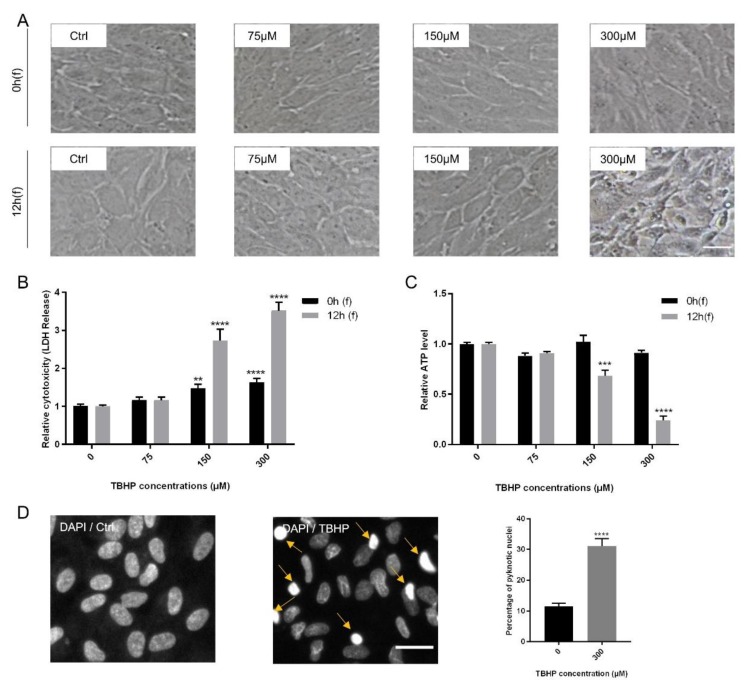
Modeling oxidative stress in ARPE19 cells using tert-butyl hydroperoxide (TBHP). Confluent ARPE19 cells were exposed to TBHP (0, 75, 150, or 300 µM) for 4 h. Changes in morphology of the cells (**A**), lactate de-hydrogenase (LDH) release (**B**) and intracellular ATP levels (**C**) were assessed at 0 h and 12 h following TBHP exposure (0 h(f) and 12 h(f), respectively). Severe morphological changes were obvious in cells treated with 300 µM TBHP at 12 h(f). Nuclear condensation was detected by DAPI staining and visualized and quantified by the operetta high content imaging system for the most effective TBHP concentration and following time (300 µM, 12 h(f)). Examples of condensed nuclei are shown by yellow arrows. The number of condensed nuclei are presented as percentage of total number of nuclei (*n* = 4). (**D**). Data are shown as mean ± S.E.M and experiments were repeated at least three times. *p*-value: <0.01 (**), < 0.001 (***), < 0.0001 (****) (One-way ANOVA, Dunnett’s multiple comparison test).

ARPE19 cells were subjected to increasing concentrations of TBHP (0, 75, 150, 300 µM) for 4 h and analyzed at two time points following (f) TBHP exposure: immediately at the end (0 h(f)) and 12 h after exposure (12 h(f)). As read-out parameters for damaged cells, morphological changes were assessed first. While treatment with TBHP at 0, 75, and 150 µM did not cause obvious changes in cell morphology, exposure to 300 µM TBHP induced apparent cellular changes 12 h after exposure including cellular shrinkage, disturbance of cell membrane, alteration from semi-hexagonal structure to asymmetrical shapes, and conversion from a monolayer to a detached and disconnected layer (Figure 1A).

To measure cell viability and cytotoxicity, lactate de-hydrogenase (LDH), ATP, and MTS assays were performed at 0 h(f) and 12 h(f). LDH is released into the cell medium due to disturbed cell membrane integrity, thus representing an early marker of cell death by apoptosis or necrosis [60]. TBHP significantly increased LDH release in a time and dose-dependent manner at 150 and 300 µM TBHP. Furthermore, the level of LDH release at 300 µM TBHP was approximately twofold higher than at 150 µM for both following time points, i.e., 0 h(f) and 12 h(f) (Figure 1B). Cell death is also associated with ATP depletion in affected cells [33,64]. Concentrations of 150 and 300 µM TBHP also significantly reduced ATP levels at 12 h(f), with the reduction by 300 µM of TBHP being threefold higher than by 150 µM (Figure 1C).

The MTS assay upon exposure of ARPE19 cells to 300 µM TBHP following 0 h(f) and 12 h(f) showed an increasing rate of cell death at 12 h(f) (63.9%) compared to controls at this time point (Supplementary Figure S1). As 300 µM of TBHP had the most significant effect on cells, this was used for all further experiments. To further assess the influence of oxidative stress caused by TBHP on ARPE19 cells, nuclear architecture and condensation of treated cells were analyzed. Chromatin condensation is a hallmark of cell death [66,67]. Therefore, nuclear staining with DAPI indicated a significant increase (3-fold) in the number of pyknotic nuclei at 12 h(f) in comparison to controls (Figure 1D). Thus, incubation with 300 µM TBHP for 4 h causes strong oxidative stress in ARPE19 cells that is robust and quantifiable 12 h after exposure.

### 2.2. Protection of Junctional Integrity and Morphology of TBHP-Stressed ARPE19 Cells by Crocetin Treatment

The saffron component crocetin represents a promising factor to counteract the detrimental effects caused by oxidative stress, as has been observed in some retinal cells [9,50]. To determine cytotoxicity of crocetin for ARPE19 cells, a cell viability assay by MTS was performed. ARPE19 cells were incubated for 24 h with different concentrations of crocetin, i.e., 0, 100 and 200 µM, and as a control for cytotoxicity of the solvent, cells were incubated with the same amount of the solvent (0.2% dimethyl-sulfoxide; DMSO). No statistically significant differences in viability were determined between crocetin-treated groups and solvent controls after 24 h (Supplementary Figure S2B). Thus, 100 µM concentration was chosen to determine whether lower concentration of crocetin is sufficient to prevent oxidative stress in TBHP-treated ARPE19 cells.

**Figure 2:**
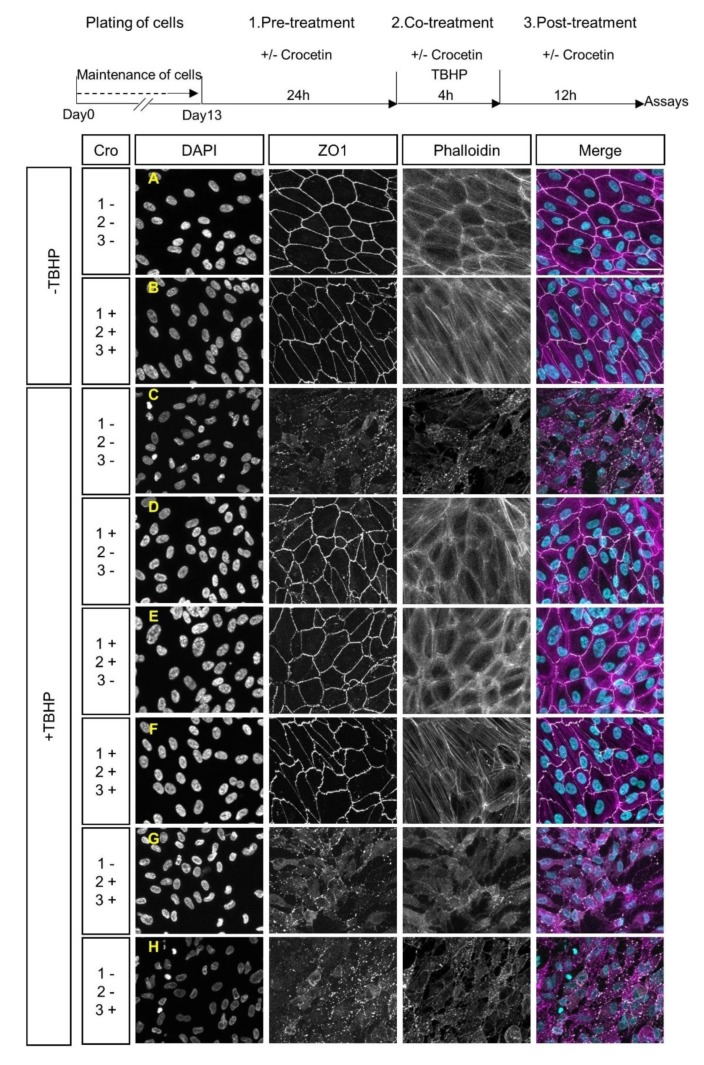
Effects of crocetin and TBHP on cellular morphology and junctional integrity. Schematic overview of crocetin treatment, which was applied at different time points. These were combinations of a 24 h pre-treatment with crocetin, 4 h co-treatment of crocetin with TBHP, and 12 h of post-treatment with crocetin in the media without TBHP. The ARPE19 cells were classified into five experimental groups based on various crocetin treatment protocols, which differed in timing of treatment with crocetin. These were combinations of pre-treatment (1.), co-treatment (2.), and post-treatment (3.) times. All of them were common in exposure to TBHP for 4 h. Images display the nuclear morphology (DAPI), cell-junction membrane marker (ZO1), F-actin distribution (Phalloidin), and co-labeling of DAPI, ZO1, and Phalloidin (Merge). The junctional integrity and cytoskeleton were preserved in all experimental groups containing pre-treatment with crocetin (**D**–**F**) to a comparable extent as in non-stressed (-TBHP) controls (**A**,**B**). In contrast, these features are severely disrupted in groups without pre-treatment with crocetin (**G**,**H**), as seen in the TBHP-only group (**C**). Scale bar: 50 µM. Cro: crocetin.

To analyze the effect of crocetin on junctional integrity and morphological features of stressed RPE cells, expression of ZO1 and F-actin was assessed by immunocytochemistry. The TBHP-exposed group (+TBHP; Figure 2C), in contrast to the non-exposed groups (-TBHP; Figure 2A,B), showed morphological changes, as evident by the disruption of the cytoskeleton and loss of ZO1 expression. ARPE19 cells were treated with crocetin at different time points including (i) 24 h before the addition of TBHP (pre-treatment), and/or (ii) 4 h in parallel with TBHP (co-treatment), and/or (iii) for 12 h after TBHP exposure (post-treatment) (See experimental scheme in Figure 2). The +TBHP groups, thus, contained pre-treatment (Figure 2D), pre-treatment + co-treatment (Figure 2E), pre-treatment + co-treatment + post-treatment (Figure 2F), co-treatment and post-treatment (Figure 2G), and post-treatment (Figure 2H). Crocetin pre-treatment prevented TBHP-induced changes in cell shape and junctional integrity (i.e., pre-treatment alone (Figure 2D), pre-treatment + co-treatment (Figure 2E), pre-treatment + co-treatment + post-treatment (Figure 2F), and resembled controls not exposed to TBHP (Figure 2A,B). However, in the non-pre-treatment groups (Figure 2G,H), TBHP-induced morphological changes were still observed, i.e., disorganization of the cytoskeleton and disruption of junctional integrity. In summary, these results showed the effectiveness of crocetin to protect ARPE19 cells from damage by oxidative stress when applied as a pre-treatment.

### 2.3. Protection of ARPE19 Cells from TBHP-induced Oxidative Stress using Crocetin Treatment

**Figure 3:**
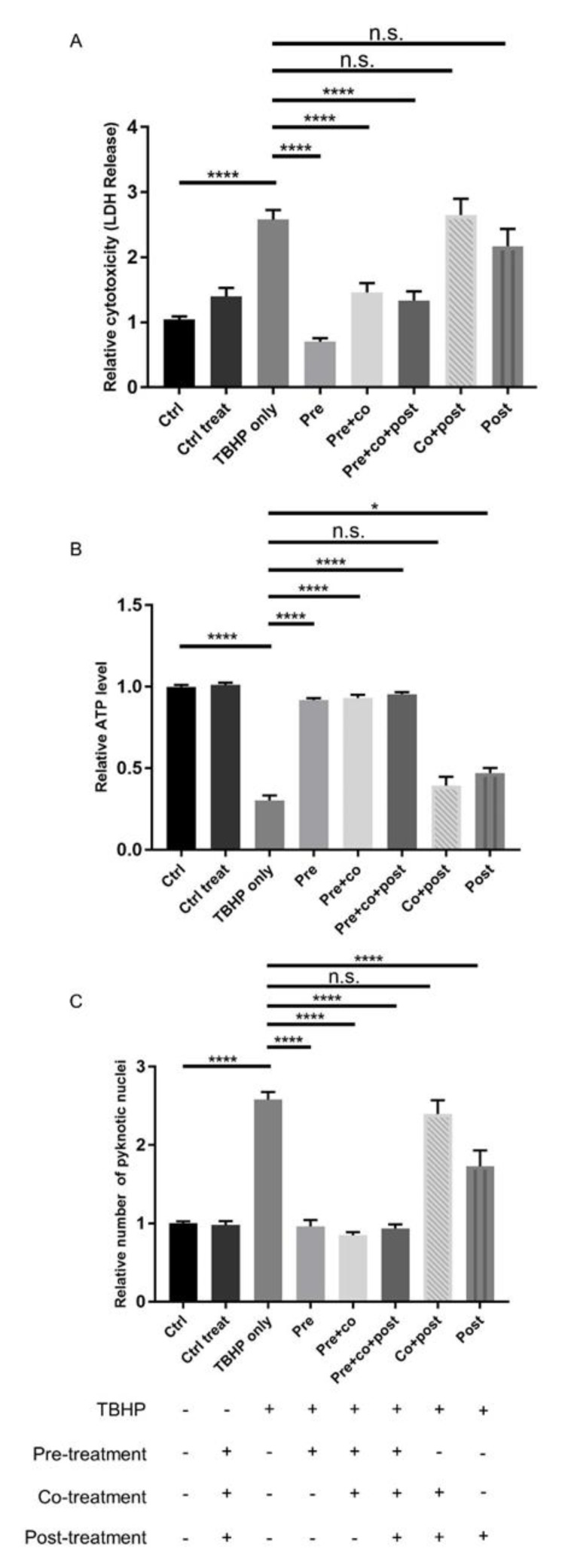
Effect of crocetin on TBHP-induced oxidative stress in ARPE19 cells. ARPE19 cells were treated with different protocols, and then LDH (**A**) and ATP (**B**) assays, as well as counting of pyknotic nuclei (**C**) were performed to assess the effect of these treatment protocols. The response of ARPE19 cells to crocetin can be categorized into two groups: pre-treated and non-pre-treated. LDH and ATP levels were preserved in pre-treated groups comparable to controls (-TBHP), whereas levels changed in non-pre-treated groups to a similar extent as the TBHP-only group. Quantification of condensed nuclei confirmed the protective action of pre-treatment with crocetin on TBHP stressed ARPE19 cells. The number of pyknotic nuclei were increased similar to the TBHP-only group, but not in pre-treated groups. Data are shown as mean ± S.E.M. *p*-value: < 0.05 (*), < 0.0001 (****) *n* = 4, (One-way ANOVA, Tukey’s multiple comparison test). n.s. = non significant.

To investigate the therapeutic potential of crocetin as a preventive or curative factor, ARPE19 cells were treated with crocetin, as shown schematically in Figure 2 and as detailed above. Cell viability and cytotoxicity assays were performed at 12 h(f). The LDH assay revealed that crocetin pre-treatment—either with or without co-treatment and post-treatment—efficiently protects ARPE19 cells against oxidative stress and subsequent cell membrane leakage (Figure 3A). Interestingly, in the non-pre-treatment groups, i.e., co-treatment and/or post-treatment, LDH release was not significantly different to the oxidative stress group (TBHP only) (Figure 3A).

Similar results were obtained by determining ATP levels and pyknotic nuclei. While intracellular ATP in TBHP exposed cells pre-treated with crocetin showed the same level as non-stressed controls, cells that had co-treatment and/or post-treatment with crocetin showed only minor increases in ATP compared to TBHP-only-treated ARPE19 cells (Figure 3B). The results of nuclear staining to determine the number of pyknotic nuclei were in accordance with the LDH and ATP results. The number of pyknotic nuclei increased in both groups of non-pre-treatment category as well as in the TBHP-only group. In contrast, the number of pyknotic nuclei were kept as low as that in non-stressed control groups in all TBHP-exposed groups with crocetin pre-treatment (Figure 3C). In summary, pre-treatment with crocetin effectively protects ARPE19 cells from damage by TBHP-induced oxidative stress.

**Figure 4:**
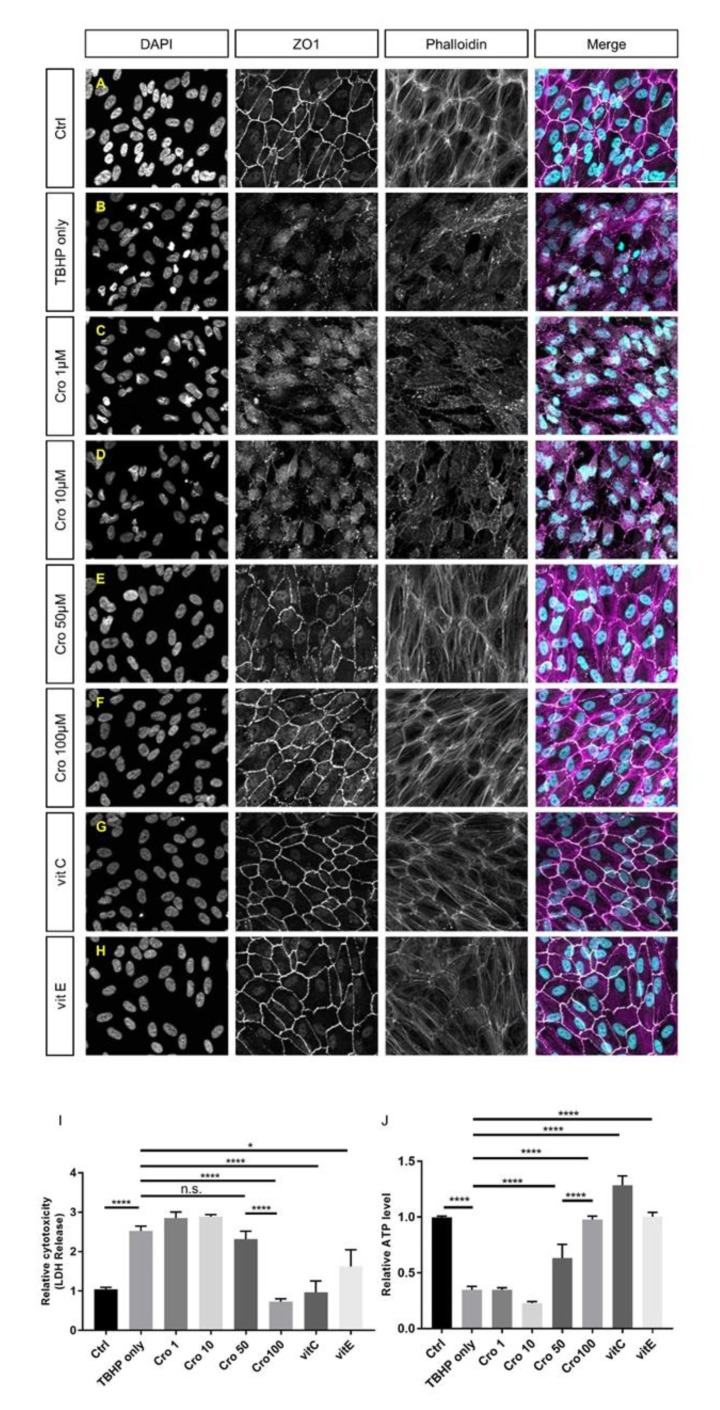
Comparison of the efficacy of different concentrations of crocetin and vitamins C and E in cellular morphology, cell viability, and intracellular ATP levels of TBHP-treated ARPE19. ARPE19 cells were pre-treated with crocetin (1, 10, 50, and 100 µM) or vitamin C and E (100 µM). After exposure to TBHP for 4 h with 12 h following time, the nuclear morphology (DAPI), junctional integrity (ZO1), and cytoskeleton (Phalloidin) were assessed by immunocytochemistry. The nuclear morphology, junctional integrity and cytoskeleton were preserved in groups, which are pre-treated with crocetin (100 µM; **F**), vitamin C (**G**) or vitamin E (**H**) to a comparable level as controls (**A**). Also, 50 µM crocetin (**E**) induced some protection against oxidative stress but not to the extent of 100 µM crocetin. In contrast, pre-treatment with 1 and 10 µM crocetin (**C**,**D**) could not protect ARPE19 cells from TBHP-induced oxidative stress and caused disruptions in cytoskeleton, junctional integrity, and nuclear morphology similar to the TBHP-only group (**B**). Additionally, the effects of pre-treatment of ARPE19 cells with crocetin (1, 10, 50, or 100 µM) and vitamins C and E (100 µM) were determined using LDH and ATP assays (I and J, respectively). In accordance with the morphological results, crocetin at concentrations of 1 and 10 µM did not protect against oxidative stress. Although, 50 µM crocetin reduced LDH (**I**) and increased ATP levels (**J**) in comparison to the TBHP-only group and lower concentrations of crocetin, it was not as effective as 100 µM crocetin or vitamins C or E. Data are shown as mean ± S.E.M. *p*-value: < 0.05 (*), < 0.0001 (****), (One-way ANOVA, Tukey’s multiple comparison test). Scale bar: 50 µM. Ctrl: Control, Cro: Crocetin, Vit: vitamin. n.s. = non significant.

To investigate which concentrations of crocetin cause protection and, additionally, to compare its effects with well-known antioxidants, TBHP-induced ARPE19 cells were pre-treated with 1, 10, 50, and 100 µM of crocetin or 100 µM of vitamin C or vitamin E, respectively (Figure 4C–H). At concentrations of 1 and 10 µM, crocetin was not able to preserve ARPE19 cells from TBHP-induced morphological changes of tight junctions, cytoskeleton, or nuclear morphology (Figure 4C,D) and the oxidative stress-induced detrimental effects were as harsh as in the TBHP-only group (Figure 4B). While signs of protection were observed using 50 µM (Figure 4E), it was not as effective as 100 µM crocetin (Figure 4F). In accordance with the morphological results, crocetin at concentrations of 1, 10, and 50 µM was unable to prevent an increase in LDH release (Figure 4I) or lead to a decrease in intracellular ATP levels (Figure 4J), though first signs of protection were observed using 50 µM crocetin. In comparison to vitamin C and E, 100 µM crocetin revealed to be effective in the protection of cell morphological parameters, i.e., disorganization of cytoskeleton, disturbance of junctional integrity, and nuclear morphology (Figure 4A–H), as well as LDH release and ATP levels (Figure 4I,J).

### 2.4. Protection of Energy Production Pathways in Stressed ARPE19 Cells by Crocetin Pre-Treatment

**Figure 5:**
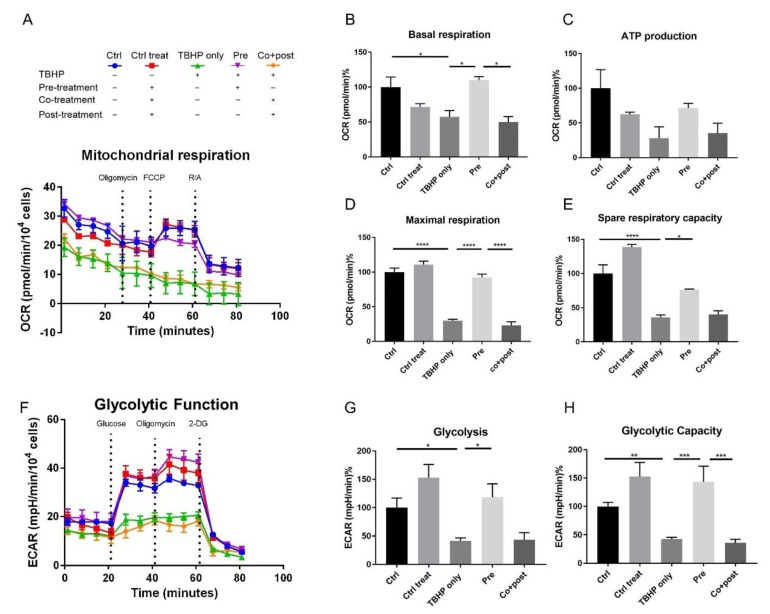
Effect of crocetin on bioenergetic function of ARPE19 cells. ARPE19 cells were pre-treated with crocetin in pre-treatment group (pre) and then all of the experimental groups except controls were exposed to TBHP for 4 h. After 12 h, oxygen consumption rate (OCR) and extracellular acidification rate (ECAR) were measured using a Seahorse XF^e^96 analyzer to determine mitochondrial respiration (**A**) and glycolytic function (**F**) over time, respectively. Parameters of mitochondrial respiration including basal respiration (**B**), ATP production (**C**), maximal respiration (**D**), and spare respiratory capacity (**E**) were reduced in TBHP-only and co-treatment+post-treatment (co+post) groups compared to control (ctrl), but crocetin pre-treatment (pre) increased all the parameters compared to TBHP-only group. For glycolytic function, glycolysis (**G**) and glycolytic capacity (**H**) were determined and showed significant reduction in TBHP-only and co+post groups, while in the pre-treatment group (pre) significantly increased similar to controls. Data are shown as mean ± S.E.M. *p*-value: < 0.05 (*), < 0.01 (**), < 0.001 (***), < 0.0001 (****) *n* = 4, (One-way ANOVA, Tukey’s multiple comparison test).

To gain further insights into the influence of oxidative stress and crocetin treatment on the bioenergetic function of ARPE19 cells, their mitochondrial and glycolytic profiles were measured using the XF^e^96 Extracellular Flux Analyzer (Seahorse Bioscience) allowing a system-view on their metabolic performance in real-time. The mitochondrial respiration of ARPE19 cells was studied using the Mito Stress Test assay. Results are shown in Figure 5A–E. Five experimental groups were analyzed: control (no TBHP, no crocetin; Figure 5A, blue), control of treatment (no TBHP, crocetin pre-, co-, and post-treatment; Figure 5A, red), TBHP-only (TBHP, no crocetin; Figure 5A, green), pre-treatment (TBHP + crocetin pre-treatment; Figure 5A, violet), co- + post-treatment (TBHP + crocetin co- and post-treatment; Figure 5A, orange).

The results of this assay confirm the protective effect of crocetin pre-treatment on the health of the stressed ARPE19 cells. As can be seen in Figure 5A, while TBHP-only and co+post-treated groups no longer responded appropriately to the injected drugs, TBHP exposed cells which were pre-treated with crocetin displayed a robust reaction similar to the control groups. Basal respiration levels, while recovered to a comparable level with controls in the pre-treatment group (110% ± 5), was severely reduced in TBHP-only (57.32% ± 9.03) and co+post-treatment (49.8% ± 8.04) groups (Figure 5B). Following basal respiration measurement, the first inhibitor (oligomycin) was injected. Oligomycin binds oligomycin sensitivity-conferring protein (OSCP) of the mitochondrial ATP synthase complex to block proton conductance across the ATP synthase complex and inhibits production of mitochondrial ATP [68,69]. As a result of disturbance in electron flow through the electron transport chain (ETC), oligomycin causes a reduction in oxygen consumption rate (OCR) related to cellular ATP production. The ATP production through mitochondrial respiration was reduced in TBHP-only (27.91% ± 16.49) and co+post-treatment (35.28% ± 14.36) groups, but was largely preserved in the pre-treatment group (71.26% ± 6.85) (Figure 5C). With the second injection, carbonyl cyanide-4 (trifluoromethoxy) phenylhydrazone (FCCP) caused a collapse in the proton gradient and a mitochondrial membrane potential disruption. As a result, OCR by complex IV reaches the maximum and maximal respiration can be calculated. This parameter in the pre-treatment group (92.16% ± 4.86) was significantly higher than TBHP-only (29.68% ± 2.34) and co+post-treatment (23.11% ± 5.04) groups. The final calculated parameter was spare respiratory capacity which is an indicator of the cells capacity to respond to increased energy demand under stress. This capacity was maintained at a higher level in the pre-treatment group (76.61% ± 0.74) in comparison to TBHP-only (35.97% ± 3.47) and co+post-treatment (40.15% ± 5.24) groups (Figure 5E). Following the third injection which was a mixture of rotenone and antimycin A (R/A), mitochondrial respiration shuts down, and the energy production shifted to glycolytic pathways.

Similar to mitochondrial respiration, in the glycolytic function graph (Figure 5F), the pre-treatment group clearly segregated with the control samples. In this assay, two parameters were calculated: glycolysis (Figure 5G) and glycolytic capacity (Figure 5H). Glycolysis is the conversion of glucose to pyruvate. This parameter was measurable after injection of a saturating concentration of glucose. This energy production pathway was completely preserved in the pre-treatment group but not in TBHP-only (41.75% ± 4.97) and co+post-treatment (43.82% ± 12.15) groups (Figure 5G). The second injection in this assay was oligomycin which shuts down oxidative phosphorylation and drives the cell to use glycolysis to its maximal capacity, thus, the glycolytic capacity can be calculated. Here, this capacity was increased in the crocetin-only control as well as in the pre-treatment groups more than in the no-treatment control (152.7% ± 25.1 and 143.5% ± 27.57, respectively). However, in the TBHP (42.68 ± 3.07) and co+post-treatment (35.9 ± 6.33) groups, the glycolytic capacity of cells was significantly decreased (Figure 5H). This indicates that crocetin may increase the glycolytic capacity of the cells independent of TBHP-treatment. The third injection was 2-DG, an analog of glucose that competes with glucose for binding to the first glycolytic enzyme, hexokinase. Therefore, a reduction in extracellular acidification rate (ECAR) confirms that produced ECAR in the experiment was a result of glycolytic function. In summary, these two energy production pathways were severely decreased in TBHP-only and co+post-treatment groups, while crocetin pre-treatment protected ARPE19 cells from these detrimental effects of oxidative stress.

### 2.5. Crocetin Protects Stressed ARPE19 Cells through ERK1/2 Activation

**Figure 6:**
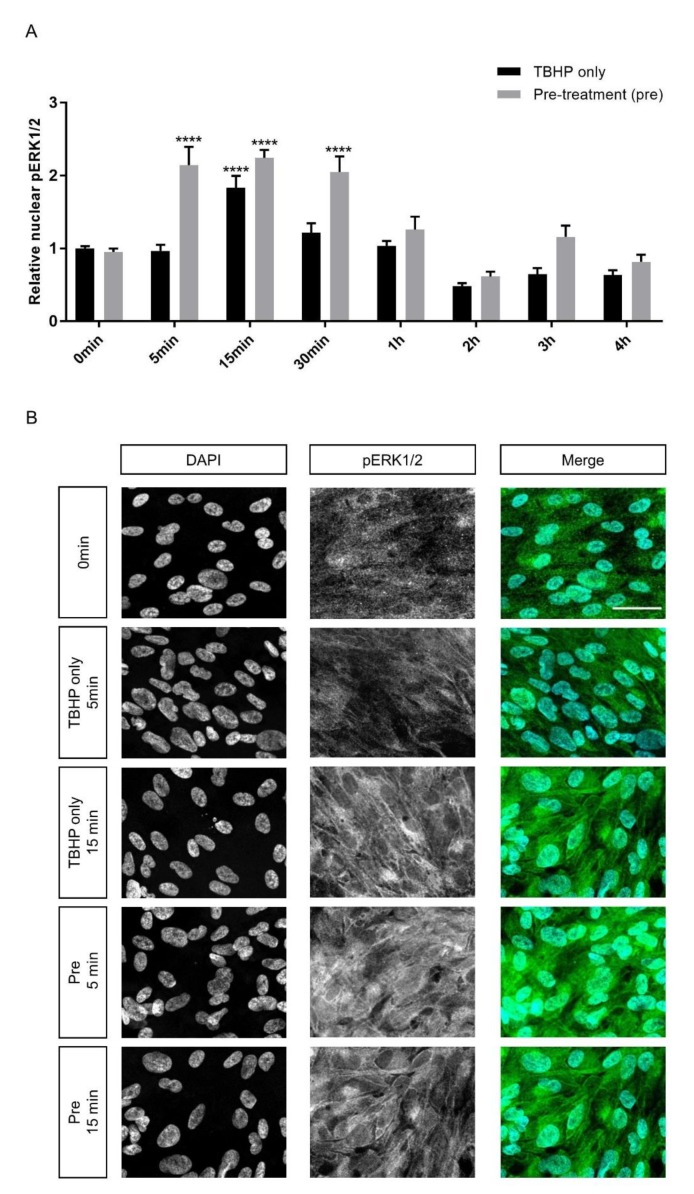
Effect of crocetin on extracellular signal-regulated kinase 1/2 (ERK1/2) activation in ARPE19 cells. ERK1/2 phosphorylation was investigated at different time points (5, 15, 30 min and 1, 2, 3, 4 h) during 4 h exposure to TBHP. ARPE19 cells were only exposed to TBHP or were additionally pre-treated for 24 h with crocetin. While ERK1/2 showed activation and nuclear translocation in the TBHP-only group at 15 min, ERK1/2 activation was observed in the pre-treatment group already at 5 min and lasted for 30 min (**A**). At 5, 15, and 30 min intervals, the activation level of ERK1/2 in the pre-treatment group was significantly and constantly higher than in the TBHP-only group (A). Thus, pre-treatment with crocetin caused activation of ERK1/2 earlier and for a longer time in comparison to TBHP-only. Immunocytochemistry results (**B**) on ARPE19 cells in controls (0 min), TBHP-only at 5 min, TBHP-only at 15min, as well as TBHP plus crocetin pre-treatment at 5 and 15 min are shown for DAPI, and phosphorylated (p) ERK1/2 (B; the third column represents the merged images of DAPI and pERK1/2). An increase in pERK1/2 signal was observed at 15 min but not at 5 min in TBHP exposed cells (in comparison to non-exposed (0 min) controls), but already at 5 min and also at 15 min in TBHP exposed cells pre-treated with crocetin. Scale bar: 40 µM. Data are shown as mean ± S.E.M. *p*-value: < 0.0001 (****) (One-way ANOVA, Dunnett’s multiple comparison test).

To investigate whether crocetin can influence cell signaling pathways as another protection mechanism in ARPE19 cells, activation of two extracellular signal-regulated kinases (ERK1/2) of the mitogen-activated protein kinase (MAPK) family were studied. Literature review suggested a range of intervals within the first hour, especially at 15 min, as the most important time points for ERK1/2 activation and nuclear translocation [24,70]. Therefore, ERK1/2 phosphorylation (p) was assessed in experimental groups in three intervals during the first hour and continued until the end of the 4 h exposure to TBHP. There was a transient peak of pERK1/2 with an increase of around 84 percent at 15 min in the TBHP-only group in comparison to control (1.84 ± 0.18 vs. 1 ± 0.03) (Figure 6A). In the crocetin pre-treatment groups, this transient activation already started at 5 min and was maintained at the 15 and 30 min time-points. The ERK1/2 activation in pre-treatment groups at these intervals was twofold higher than at baseline. The activation of ERK1/2 was significantly higher in the pre-treatment group than TBHP-only at 5 (*p*-value: < 0.0001) and 30 min (*p*-value: 0.0003). Even at 15 min the phosphorylation level of ERK1/2 is about 40 percent greater in the pre-treatment group in comparison to TBHP-only. At other intervals including 1, 2, 3, and 4 h, there was no significant difference between TBHP-only and pre-treatment groups and overall pERK1/2 levels decreased. The immunocytochemistry results of 5 and 15 min for TBHP-only and pre-treatment groups are shown in Figure 6B. To detect whether expression level of ERK1/2 was changed, the cellular signal of ERK1/2 was measured in these same groups and time points. Data of whole cell signals of ERK1/2 (Supplementary Figure S4) revealed that the ERK1/2 expression level was not changed over the course of the experiment, and there were no significant differences between TBHP-only and pre-treatment groups. This indicates that the differences in pERK1/2 are due to the crocetin pre-treatment rather than differences in basal expression levels of ERK1/2.

## 3. Discussion

Diminishment of the antioxidant defense system results in accumulation of ROS in aged RPE cells and subsequently leads to degeneration of RPE cells and the onset of AMD [71,72]. It has been shown that the degeneration of RPE cells is partly prevented using natural anti-oxidant compounds due to both oxidative stress reduction and antioxidant defense system promotion [28,73,74,75,76]. Crocetin, one of the main extracts of saffron, is a natural anti-oxidant with robust anti-oxidative characteristics [52,53]. In this study, the protective effect of crocetin against TBHP-induced oxidative stress is consistent with the impact of saffron in six clinical trials related to AMD. In these clinical trials, positive effects of saffron on patients vision were reported by significant improvements in objective (ERG) and subjective measures (Snellen, LogMar, EDTRS charts) of visual acuity [42,43,44,45,46,47] and increase in contrast sensitivity (CS) [46]. However, the mechanism and pathways involved in the beneficial effects of saffron on retinal tissues have not been fully identified.

Although recent studies provided evidence that pre-treatment with saffron/crocin protects retinal cells from damage, to the best of our knowledge, no study has reported the effects of crocetin on RPE cells. Crocetin can pass through the blood–retinal-barrier to affect the RPE as well as the neural retina [50], thus representing a potential candidate for the protection of AMD related cellular damage. Our results indicate a crucial role of pre-treatment with crocetin in protection against TBHP-induced oxidative stress. Crocetin pre-treatment caused the preservation from lipid peroxidation, cell membrane damage and release of cytosolic contents, loss of ZO1 expression, and cytoskeletal disorganization. Moreover, decrease in the number of pyknotic nuclei and increase in intracellular ATP levels were indicative of protection against cell death.

Cells utilize two energy production pathways, oxidative phosphorylation within mitochondria and glycolysis within the cytosol [77]. In RPE cells, which have high metabolic demand and number of mitochondria [23], abnormal activity or damage of mitochondria contributes to the increase in ROS production with aging [78]. Therefore, mitochondria of RPE cells represent a primary site in AMD pathology and can potentiate cell death [79]. Several studies support the idea of mitochondria dysfunction in AMD by showing disrupted mitochondria architecture, numbers, mass, protein expression as well as mtDNA damage [79,80]. Our obtained results demonstrate that both pathways are negatively affected by TBHP treatment, but can be fully protected via pre-treatment with crocetin. We used the seahorse XF^e^96 analyzer to measure real-time changes in OCR and ECAR in response to effectors and inhibitors of glycolysis and mitochondrial activity in one pre-treated and one non-pre-treated group and compared these to non-treated controls and TBHP-exposed cells. We observed that TBHP-induced oxidative stress contributes to mitochondrial dysfunction, which was reflected in a reduction in metabolic capacity of ARPE19 cells in all parameters including basal respiration, ATP production, maximal respiration, and spare respiratory capacity. The increase in ECAR after oligomycin addition is indicative of a shift to ATP production by glycolysis. In stressed ARPE19 cells of TBHP-only and co+post groups, this increase was not observed and was dramatically lower than in control groups. In contrast to these stressed groups, crocetin pre-treatment maintained the glycolytic capacity of RPE cells and improved the functionality of RPE cells, as seen by their subsequent response to glycolysis stress test reagents. In summary, crocetin preserved the metabolic function of mitochondria and kept the glycolysis pathway active in the pre-treatment group to serve as a backup for energy production. Therefore, our results provide evidence that one mechanism of crocetin action in preventing oxidative stress is through protecting both, i.e., mitochondrial and glycolytic, energy production pathways. Our above-mentioned results for metabolic function of stressed and crocetin-treated ARPE19 cells, are consistent with studies that showed RPE from AMD donors exhibit reduced mitochondrial and glycolytic function compared to healthy donors [81] and also confirm the suggestion of using therapeutic approaches to target RPE mitochondria as an effective strategy for AMD treatment [79,80,81,82,83]. However, our results for ATP production via glycolysis are in contrast to some studies that displayed an increase in glycolysis in RPE of AMD donors [78] and human stem cell-derived RPE cells stressed with Paraquat [84].

The mitogen activated protein kinase (MAPK) cascades are involved in regulation of many cellular processes, including cell proliferation, development, migration, survival, and cell death [85]. Our data demonstrate the activation of ERK1/2 in both TBHP and crocetin pre-treated groups within 15 min of TBHP exposure. The activation of ERK in both of these groups can be related to promotion of survival pathways. The trend of cytoplasmic (Supplementary Figure S3) and nuclear (Figure 6A) ERK1/2 activation were similar, indicating that the activation of ERK1/2 may begin prior to its nuclear localization, consequently leading to activation of survival and proliferation pathways [70,86]. Furthermore, we did not observe cytoplasmic accumulation of activated ERK1/2 (Supplementary Figure S3), which is proposed to activate cytoplasmic pro-apoptotic proteins, such as death associated protein kinase (DAPK), Bik, and block nuclear translocation [70]. Second, prolonged activation can promote pro-apoptotic effects [87], while transient activation of ERK preserve from cell death [88]. The transient activation of ERK1/2 in both TBHP-only and pre-treatment groups in the first minutes of TBHP exposure potentially induce survival pathways activation. However, the short-term activation in the TBHP group at 15 min after TBHP exposure may be insufficient to protect the RPE from cell damage. In contrast, the earlier and increased activation seen in crocetin pre-treated groups, i.e., the activation of pERK1/2 and its nuclear localization had already started 5 min following TBHP exposure and was stably maintained for 30 min, might promote the protection against cell death activation.

Regarding the involvement of ERK1/2 in cell survival, there are conflicting results in the literature as to whether ERK phosphorylation enhancement adversely or favorably influences cell survival in RPE cells [24]. Chong and Zheng showed that an anti-malaria drug (Artemisinin) that activated ERK/CREB survival signaling pathway in the D407 RPE cell line, failed to repress cell death in the presence of an ERK inhibitor, PD98059 [27]. However, Glotin et al., determined transient activation of ERK1/2 in response to TBHP exposure and nuclear accumulation of its inactive form in ARPE19 cells induces cell death. Therefore, inhibition of the MEK-ERK module can block apoptosis [89]. In regards to crocetin and its effect on the ERK1/2 pathway, it has been shown that the mechanism of its protective action depends on the cell type. For instance, its protective effect against cancer [90], cardiac hypertrophy [56], and atherosclerosis [91] is through inhibition of ERK1/2 activation. In RPE cells, it has been shown that in response to platelet-derived growth factor (PDGF) induced proliferation and migration, crocetin inhibits ERK in a time and dose-dependent manner [92], but there has, thus far, been no study to investigate the effect of crocetin on ERK1/2 activation in RPE cells in response to oxidative stress. Here, we suggest that the therapeutic potential of crocetin is because of enhanced ERK1/2 activation and subsequent triggering of cell survival pathways. In another study, the effect of another main extract of saffron, crocin, was assessed on H_2_O_2_–induced oxidative stress, and the results denoted the inhibition of other MAPK family members including JNK and P38 [48]. Therefore, the MAPK pathways might be one of the signaling pathways through which saffron extracts exert their influence on cell survival, however, the effect of each extract could be different based on treatment protocol and type of oxidant.

Due to the increasing prevalence of AMD in industrialized societies with no established treatments available for dry AMD, representing approximately 90% of AMD patients, the promotion of the antioxidant defense system might represent a promising therapeutic option [30], with crocetin being an interesting candidate for its antioxidant potential. Crocetin has been shown to exhibit strong antioxidant effects including scavenging of free radicals [91], activating antioxidant enzymes, such as GSH S-transferase and GSH peroxidase [51], suppressing lipid peroxidation [56], and enhancing oxygen diffusivity through liquids, such as plasma [51]. Here, in addition to its anti-oxidant potential for suppressing lipid peroxidation that resulted in protection of cell membrane and junctional integrities, we showed further aspects of its action for oxidative stress protection. Crocetin can affect two major cellular energy production pathways and in this way support cells energetically against energy demands under oxidative situation. Furthermore, crocetin can cause protective effects by triggering MAPK-dependent survival pathways. In line with our observation, that the protective role of crocetin is particularly effective when given as a pre-treatment, i.e., before the addition of the damaging substance (TBHP), first trials using short-term saffron supplementation reported improved retinal flicker sensitivity, contrast sensitivity, and decrease in central macular thickness, in patients suffering from early AMD [42,45,46]. The protection caused by pre-treatment with crocetin is in a similar range as established antioxidants, such as vitamins C and E. Interestingly, vitamins C and E were shown to be useful for suppression of macular degeneration progression in AMD patients [93] or retinal oxidative stress models [94,95] and have been used in other studies to compare the efficiency of specific flavonoids in protection of RPE cells from oxidative stress [28]. Thus, further studies might assess whether crocetin in combination with other protective reagents, such as vitamins C or E, shows improved beneficial effects.

Several questions are still to be answered to assess the full potential of crocetin as a protectant for stressed RPE cells, e.g., what is the effect of crocetin in other oxidative stress or AMD models, including human primary or iPSC-derived RPE from healthy and AMD individuals, besides in vivo animal models with RPE degeneration?. While most studies suggest saffron as a therapeutic candidate for AMD treatment, our results provide evidence that crocetin is effective as a prevention and not a treatment after the incidence of major disease symptoms.

## 4. Materials and Methods

### 4.1. ARPE19 Cell Culture

The human RPE cell line ARPE19 was cultured in basal DMEM/F12 medium (Life Technologies, Darmstadt, Germany) supplemented with 10% FBS (Sigma–Aldrich, Taufkirchen, Germany) and 1% antibiotic-antimycotic (Life Technologies, Darmstadt, Germany) at 37 °C in a humidified 5% CO2 incubator. ARPE19 cells spontaneously obtain differentiated morphology and markers over multiple passages, so the cells in high passage number (passage 23-32) were used for all experiments to ensure cell uniformity and reproducibility. Cells were seeded in 96-well plates at a density of 1 × 10^5^ cells/well and maintained for two weeks after seeding before performing experiments. The culture medium was changed three times per week.

### 4.2. Exposure of ARPE19 Cells to TBHP As An in Vitro Model of Oxidative Stress

Two weeks after seeding of ARPE19 cells in 96-well plates, TBHP was added for 4 h in four concentrations (0, 75, 150, and 300 µM). TBHP was diluted in culture medium to a concentration of 3 mM and the desired concentrations were prepared from this stock-solution. Directly after end of exposure time, ARPE19 cells received fresh medium and were cultured for an additional 12 h before further use.

### 4.3. MTS Assay

For assessing cell viability, the MTS assay was used following the protocol provided by the company (CellTiter 96^®^ AQueous, Promega, Walldorf, Germany). In brief, cells were incubated with MTS diluted in culture medium for 2.5 h and the absorbance was measured at 490 nm by a microplate reader (FlexStation3 Multi-Mode, Molecular Devices, San Jose, CA, USA). Data were normalized to the mean value of the control group.

### 4.4. Crocetin and Vitamins Solution Preparation and Treatment

Crocetin (Toronto Research Chemicals, North York, ON, Canada) was dissolved in DMSO (AppliChem, Darmstadt, Germany) to a concentration of 90 mM and kept at -20 °C. For experiments, the stock solution was diluted with culture medium to a final concentration of 1, 10, 50, 100, or 200 µM crocetin, also diluting DMSO to a safe range (0.1–0.2%, i.e., < 0.5% (*v*/*v*)). Cells were exposed to crocetin for different incubation times including: (i) for 24 h before exposure to TBHP (pre-treatment), (ii) and/or for 4 h with TBHP (co-treatment), (iii) and/or for 12 h after exposure to TBHP (post-treatment). Ascorbic acid (vitamin C) (Sigma–Aldrich, Taufkirchen, Germany) was dissolved in water and then diluted in culture medium to a concentration of 100 µM. DL-α-tocopherol acetate (vitamin E) (Sigma–Aldrich, Taufkirchen, Germany) was diluted in water to a concentration of 10 mM, then diluted in culture medium to a concentration of 100 µM.

### 4.5. LDH Assay

Leakage of lactate dehydrogenase (LDH), a stable cytosolic enzyme, into the medium reflects cell membrane damage. The CytoTox 96^®^ Non-Radioactive Cytotoxicity Assay Kit (Promega, Walldorf, Germany) was used to determine the amount of LDH released into the medium. Cell cultures were incubated according to the manufacturer’s instructions; in brief, after treatment of ARPE19 cells, supernatant samples were transferred to a 96-well plate and equal volume of kit reagent was added to each well and incubated for 30 min at room temperature. Stop solution was added and absorbance was measured at 490 nm using a microplate reader (FlexStation3 Multi-Mode, Molecular Devices, San Jose, CA, USA). Data were normalized to the mean value of the control group.

### 4.6. ATP Assay

ATP levels represent a read out for the status of cells, as the concentration of ATP declines rapidly when cells are damaged and undergo necrosis or apoptosis. The ATPlite 1 step luminescence ATP detection Kit (PerKinElmer LAS, Rodgau, Germany) was used according to manufacturer’s instructions; in brief, after equilibration at room temperature, equal volume of ATPlite 1 step reagent was added to each well and the fluorescence signal was quantified using a luminescence reader (FlexStation3 Multi-Mode, Molecular Devices, San Jose, CA, USA). Data were normalized to the mean value of the control group.

### 4.7. DAPI Staining

After treatment, ARPE19 cells were washed 3× 5 min with PBS, fixed with 4% Paraformaldehyde for 20 min at room temperature, washed 3× 5 min with PBS and stained with DAPI (1/15000, AppliChem, Darmstadt, Germany) at 4 °C for 12 h. After washing three times with PBS (5 min), the nuclei were visualized and the number of healthy and pyknotic nuclei were quantified using the Operetta high-content imaging system (PerKinElmer, Waltham, MA, USA).

### 4.8. Immunocytochemistry

#### 4.8.1. Detection of ZO1 and F-actin

After treatments, ARPE19 cells were washed 3× 5min with PBS, fixed with 4% Paraformaldehyde for 20 min, washed 3× 5min with PBS, incubated with PBS containing Glycin 200 mM and Triton X100 (0.3%) for 20 min, and incubated in blocking solution (0.5% BSA and 0.3% Triton X100 in PBS) for 1 h. For ZO1 detection, cells were incubated with primary antibody (mouse anti-ZO1, 1:200, BD Bioscience, San Jose, CA, USA) in blocking solution for 12 h in dark at 4 °C, washed 3× 5min with PBS, followed by incubation with anti-mouse secondary antibody (goat anti-mouse DyLight488, 1:1000, Invitrogen, Karlsruhe, Germany). For visualization of F-actin, cells were incubated in AlexafluorTM 633 Phalloidin (1:100; Invitrogen, Karlsruhe, Germany). After staining, cells were incubated with DAPI for 2 h, washed with PBS containing 0.3% Triton X100 three times for 15 min, embedded in a humidified dark chamber, and imaged using a fluorescent microscope (ApoTome2 fluorescence microscope, Carl Zeiss Microscopy, Jena, Germany).

#### 4.8.2. MAPK Data Evaluation

An object-based protocol was developed to determine both nuclear and cytoplasmic distribution of ERK/pERK containing two steps: a nuclei segmentation for DAPI channel and a cell segmentation using the ERK/pERK signal. The nuclei segmentation was implemented as a ImageJ/Fiji [96] macro containing three main stages; creating 2D projection of z-stacks, pre-processing to reduce the noise contribution and object segmentation using a watershed method. In the first stage, the z-stacks were imported using Bio-Format Importer [97]. A stack project plugin, called Extend Depth-of-Field (EDF) was subsequently employed to compute an in-focus image per channel (Supplementary Figures S5 and S6) [98]. The in-focus images are supposed to have the largest number of fine structures. EDF utilized complex wavelet transform to consistently select the in focus pixels from the slices and project them on a 2D in-focus image. To assess the fine structures, Fourier analysis was conventionally applied; however, Fourier-based analysis does not provide any spatial information. On the other hand, wavelet transform provides spatial analysis of fine details.

The EDF algorithm stores a map (M) of the number of the slice per pixel corresponding to the in-focus 2D image. Unlike maximum intensity projection that is solely based on the axial distribution of the intensity, the EDF applies selection rules based on the spatial distribution. The selection rule considered the neighboring slice number in a 3 × 3 window in M: Given that M(n,m) is a pixel in M that is from slice j but most of its surrounding pixels in 3 × 3 window in M are from slice k, then the M(n,m) = j is changed to M(n,m) = k [96]. In other words, the neighboring pixels in the in-focus image are consistently checked to avoid mis-projection of pixels from above/below slice. Although this method was first developed for processing wide-field images, these selection rules made the EDF a suitable method to compensate the curvature of the bottom of the wells in confocal images.

Pre-processing included Gaussian Blur filtering to remove noise with a sigma of 4, setting the auto threshold using “Li” algorithm, and finally converting the threshold map to a mask. A distance transform watershed segmentation was then applied to segment the nuclei. Per each z-stack, one mask and one EDF image per channel were saved to be used as an image set in further analysis using CellProfiler [99]. Cell segmentation was performed using CellProfiler (version 3.1.9). This open source software provided an improved method to identify cell borders. The mask of segmented nuclei was converted from an image to the objects and subsequently the nuclei that were touching the border of the images were filtered out. The cell segmentation was, therefore, performed on the Identify Secondary Objects module using a combination of seeded watershed based on segmented nuclei from previous steps, adaptive “Otsu” three classes thresholding to suppress the background, and the Propagation method to identify the border of the dividing lines between the cells. The Measure Image Intensity modules were employed to determine the mean gray value of ERK/pERK encapsulated in nuclei, cytoplasm, and whole cell per image accordingly. The results were then saved and exported as a TXT file to be further analyzed as described in Section 4.10. The ImageJ/Fiji macro and CellProfiler pipeline are available at (Supplementary Files 1 and 2) with annotation in each step.

### 4.9. Real-time Metabolic Analysis

Mitochondrial respiration and glycolytic capacity of cells were measured using an XF^e^96 Extracellular Flux Analyzer (Agilent Technologies, Santa Clara, CA, USA). ARPE19 cells were seeded in special Seahorse 96-well plates at a density of 3.2 × 10^4^ cells/well. Different drug application protocols were applied to determine the effect of crocetin on the metabolic capacity of ARPE19 cells. A group with only pre-treatment and a group without pre-treatment but with co- and post-treatment were studied as a candidate for crocetin pre-treated and non-pre-treated groups, respectively. Similar to other assays, there were other groups additionally to compare the effects of crocetin treatment at different time points, including controls and TBHP-only exposed groups. Two days prior to performing the assay, the candidate group for pre-treatment was treated with crocetin. One day prior the assay, test groups were exposed to TBHP for 4 h and then rested in the desired condition for 12 h. The sensor cartridge was also hydrated using calibration buffer. On the day of the experiment, after washing with PBS, all of the groups were incubated with Seahorse assay medium supplemented with 17.5 mM glucose, 1 Mm glutamine, 1 mM sodium pyruvate for the Mito Stress Test and 1 mM glutamine for the Glycolysis Stress Test, pH 7.4 in 37 °C without CO_2_ for 45 min. Changes in the oxygen concentration and pH were measured as oxygen consumption rate (OCR) and extracellular acidification rate (ECAR), respectively. In the Mito Stress Test, OCR measurements for basal respiration were followed by sequential injection of oligomycin (complex V inhibitor, 2.5 µM), carbonyl cyanide-4 (trifluoromethoxy) phenylhydrazone (FCCP) (mitochondrial respiration uncoupler, 1 µM), and a mixture of rotenone (complexI inhibitor) and antimycin A (complexIII inhibitor, each 1 µM). In the Glycolysis Stress Test, base line ECAR measurements were followed by sequential injection of glucose (saturating concentration of glucose, 10 mM), oligomycin (complex V inhibitor, 1 µM), 2-deoxy-glucose (2-DG) (glucose analog, glycolysis inhibitor, 50 mM). Data were normalized by total number of cells per well quantified by DAPI staining. Minimum rate measurement after rotenone/antimycin A injection was defined as non-mitochondrial OCR. Basal respiration was calculated by subtracting non-mitochondrial OCR from the last rate measurement before first injection. Maximal respiration was calculated by the subtraction of non-mitochondrial respiration from the maximum rate measurement after FCCP injection. ATP production was measured by subtracting the minimum rate measurement after oligomycin injection from the last rate measurement before oligomycin injection. Lastly, spare respiratory capacity was calculated as a percentage by ratio of maximal respiration to basal respiration multiplied by 100. After calculation of all the above-mentioned parameters, they were represented as percent from control in their related graphs. For Glycolysis Stress Test, glycolysis and glycolytic capacity were calculated by measurement of ECAR. Glycolysis was calculated by subtracting the last rate measurement before glucose injection from the maximum rate measurement before oligomycin injection. Glycolytic capacity was calculated by subtracting the last rate measurement before glucose injection from the maximum rate measurement after oligomycin injection. Similar to Mito Stress Test, these parameters were represented as percent from control in their related graphs.

### 4.10. Statistical Analysis

Statistical analysis was performed using GraphPad Prism 7.0 statistical software (GraphPad software, Inc., San Diego, CA, USA). All experiments were analyzed using one-way ANOVA followed by a Tukey’s post-hoc test, where all groups were compared against each other, or a Dunnett’s multiple comparison post-hoc test, where all test groups were compared to the control sample. *p* < 0.05 was considered statistically significant. Data are represented as mean ± SEM.

## 5. Conclusions

Pre-treatment with crocetin preserves ARPE19 cells from TBHP-induced oxidative stress hallmarks including LDH release, ATP depletion, loss of cytoskeleton, and junctional integrity and nuclear condensation through protection of cellular energy production pathways and activation of ERK1/2 pathways.

## Supplementary Materials

Supplementary Materials can be found at https://www.mdpi.com/1422-0067/21/8/2949/s1.

## Abbreviations

AMD	Age related macular degeneration
RPE	Retinal pigment epithelium
ERK	Extracellular signal-regulated kinase
pERK	Phospho extracellular signal-regulated kinase
MAPK	Mitogen activated protein kinase
LDH	Lactate dehydrogenase
ATP	Adenosine triphosphate
ROS	Reactive oxygen species
VEGF	Vascular endothelial growth factor
GA	Geographic atrophy
TBHP	Tert-butyl hydroperoxide
DMSO	Dimethyl-sulfoxide
ZO1	Zonula occludens
ETC	Electron transport chain
FCCP	Carbonyl cyanide-4 (trifluoromethoxy) phenylhydrazone
OCR	Oxygen consumption rate
ECAR	Extracellular acidification rate
EDTRS	Early Treatment Diabetic Retinopathy Study
ERG	Electroretinography
MtDNA	Mitochondrial DNA
DAPK	Death associated protein kinase
PDGF	Platelet derived growth factor
H_2_O_2_	Hydrogen peroxide
GSH	Glutathione
JNK	*c-jun N-terminal kinase*
iPSC	Induced pluripotent stem cells
PBS	Phosphate-buffered saline
EDF	Extend Depth-of-Field
2-DG	2-deoxy glucose
Mito	Mitochondrial
CS	Contrast sensitivity
Pre	Pre-treatment
Co	Co-treatment
Post	Post-treatment
Cro	Crocetin
