# Correction: Karimi, P., et al. Crocetin Prevents RPE Cells from Oxidative Stress through Protection of Cellular Metabolic Function and Activation of ERK1/2. *Int. J. Mol. Sci.* 2020, *21*, 2949

**DOI:** 10.3390/ijms22010244

**Published:** 2020-12-29

**Authors:** Padideh Karimi, Ali Gheisari, Sylvia J. Gasparini, Hossein Baharvand, Faezeh Shekari, Leila Satarian, Marius Ader

**Affiliations:** 1 CRTD/Center for Regenerative Therapies Dresden, Center for Molecular and Cellular Bioengineering, Technische Universität Dresden, 01307 Dresden, Germany; padideh.karimi_sejzei@mailbox.tu-dresden.de (P.K.); Sylvia.gasparini@tu-dresden.de (S.J.G.); 2 CMCB/Center for Molecular and Cellular Bioengineering, Technische Universität Dresden, 01307 Dresden, Germany; ali.gheisari@tu-dresden.de; 3 Department of Stem Cell and Developmental Biology, Cell Science Research Center, Royan Institute for Stem Cell Biology and Technology, ACECR, Tehran 1665659911, Iran; baharvand@royaninstitute.org (H.B.); faezehshekari@gmail.com (F.S.); 4 Department of Developmental Biology, University of Science and Culture, Tehran 1665659911, Iran; 5 Department of Brain and Cognitive Science, Cell Science Research Center, Royan Institute for Stem Cell Biology and Technology, ACECR, Tehran 1665659911, Iran

The authors wish to make the following correction to Figure 6B of this article [1]: the merge image of group “Pre 15min” (row 5, column 3) was also added by mistake as the merge image of group “TBHP only 15min” (row 3, column 3). The images of the DAPI and pERK1/2 single channels are correct for all experimental groups. The correct merge image has been added for “TBHP only 15min” (row 3, column 3) (Figure 1).

The authors would like to apologize for any inconvenience caused to the readers by this change.

## Figures and Tables

**Figure 1:**
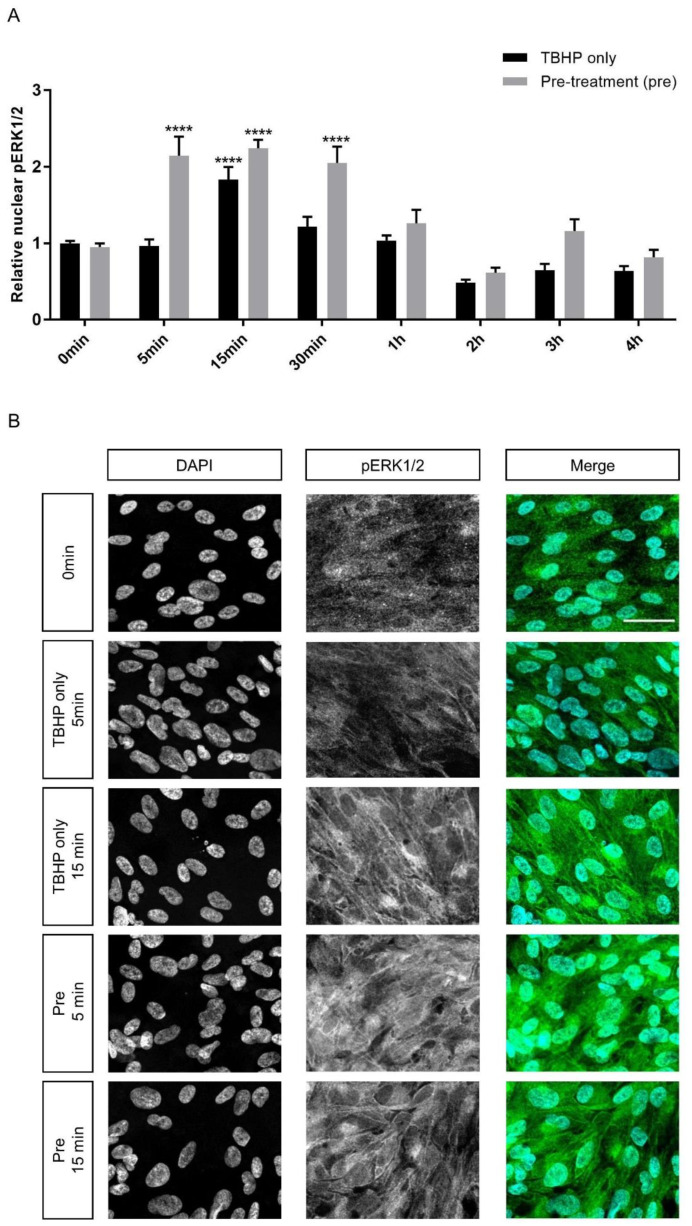
Effect of crocetin on extracellular signal-regulated kinase 1/2 (ERK1/2) activation in ARPE19 cells. ERK1/2 phosphorylation was investigated at different time points (5, 15, 30 min and 1, 2, 3, 4 h) during 4 h exposure to TBHP. ARPE19 cells were only exposed to TBHP or were additionally pre-treated for 24 h with crocetin. While ERK1/2 showed activation and nuclear translocation in the TBHP only group at 15 min, ERK1/2 activation was observed in the pre-treatment group already at 5 min and lasted for 30 min (**A**). At 5, 15, and 30 min intervals, the activation level of ERK1/2 in the pre-treatment group was significantly and constantly higher than in the TBHP-only group (**A**). Thus, pre-treatment with crocetin caused activation of ERK1/2 earlier and for a longer time in comparison to TBHP-only. Immunocytochemistry results (**B**) on ARPE19 cells in controls (0 min), TBHP-only at 5 min, TBHP-only at 15 min, as well as TBHP plus crocetin pre-treatment at 5 and 15 min are shown for DAPI, and phosphorylated (p) ERK1/2; the third column represents the merged images of DAPI and pERK1/2. An increase in pERK1/2 signal was observed at 15 min but not at 5 min in TBHP-exposed cells (in comparison to non-exposed (0 min) controls), but already at 5 min and also at 15 min in TBHP-exposed cells pre-treated with crocetin. Scale bar: 40 µM. Data are shown as mean ± S.E.M, *p*-value: <0.0001 (****) (one-way ANOVA, Dunnett’s multiple comparison test).
