# Supplementary figures and images for: Crocetin Prevents RPE Cells from Oxidative Stress through Protection of Cellular Metabolic Function and Activation of ERK1/2
Source: Int J Mol Sci. 2020 Apr 22;21(8):2949. doi: 10.3390/ijms21082949 (PMC7215651; doi:10.3390/ijms21082949)

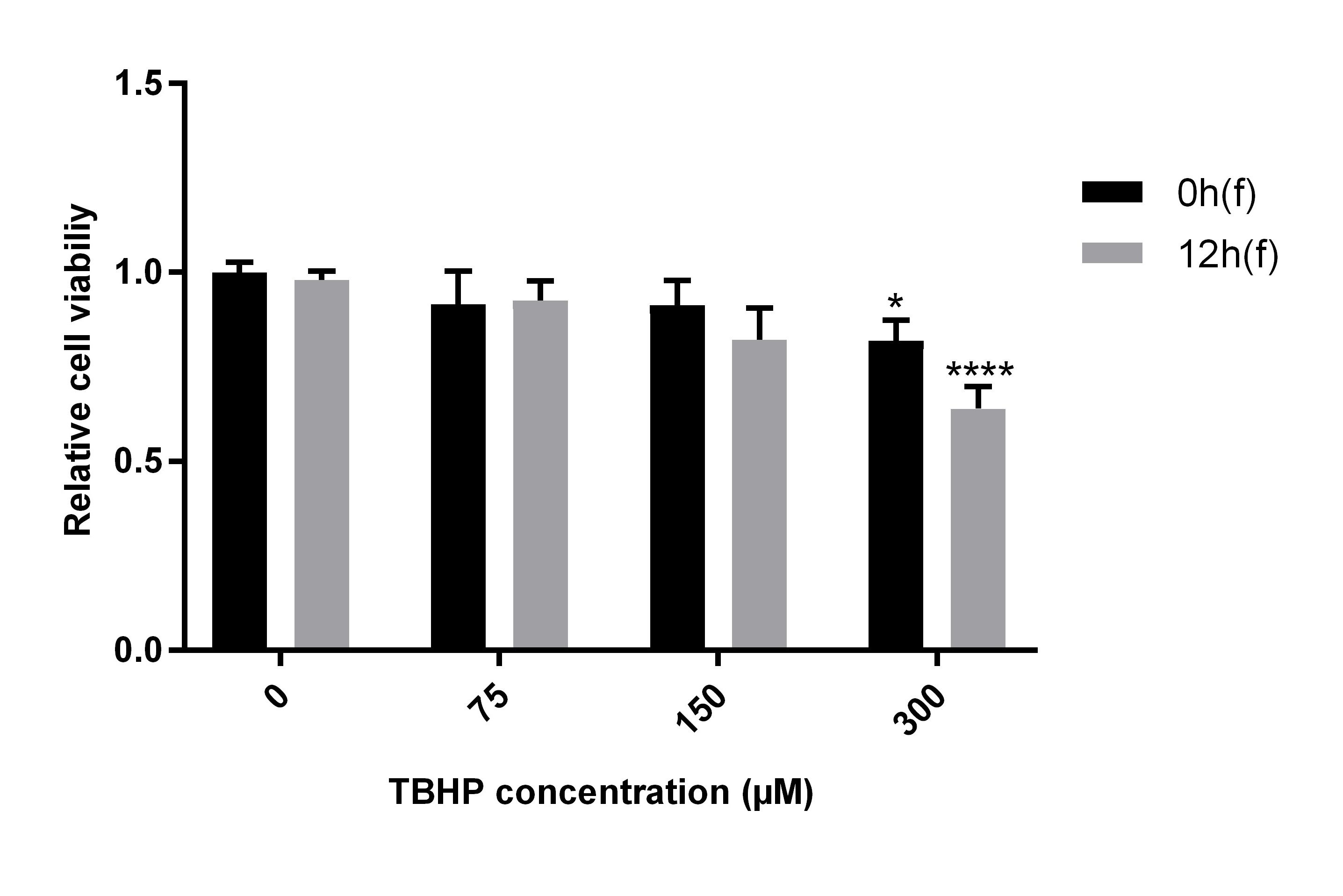

Supplement: Supplementary file 1 [file ijms-21-02949-s001.zip › supplementary files/Suupl. Figures/Supp1.jpg]

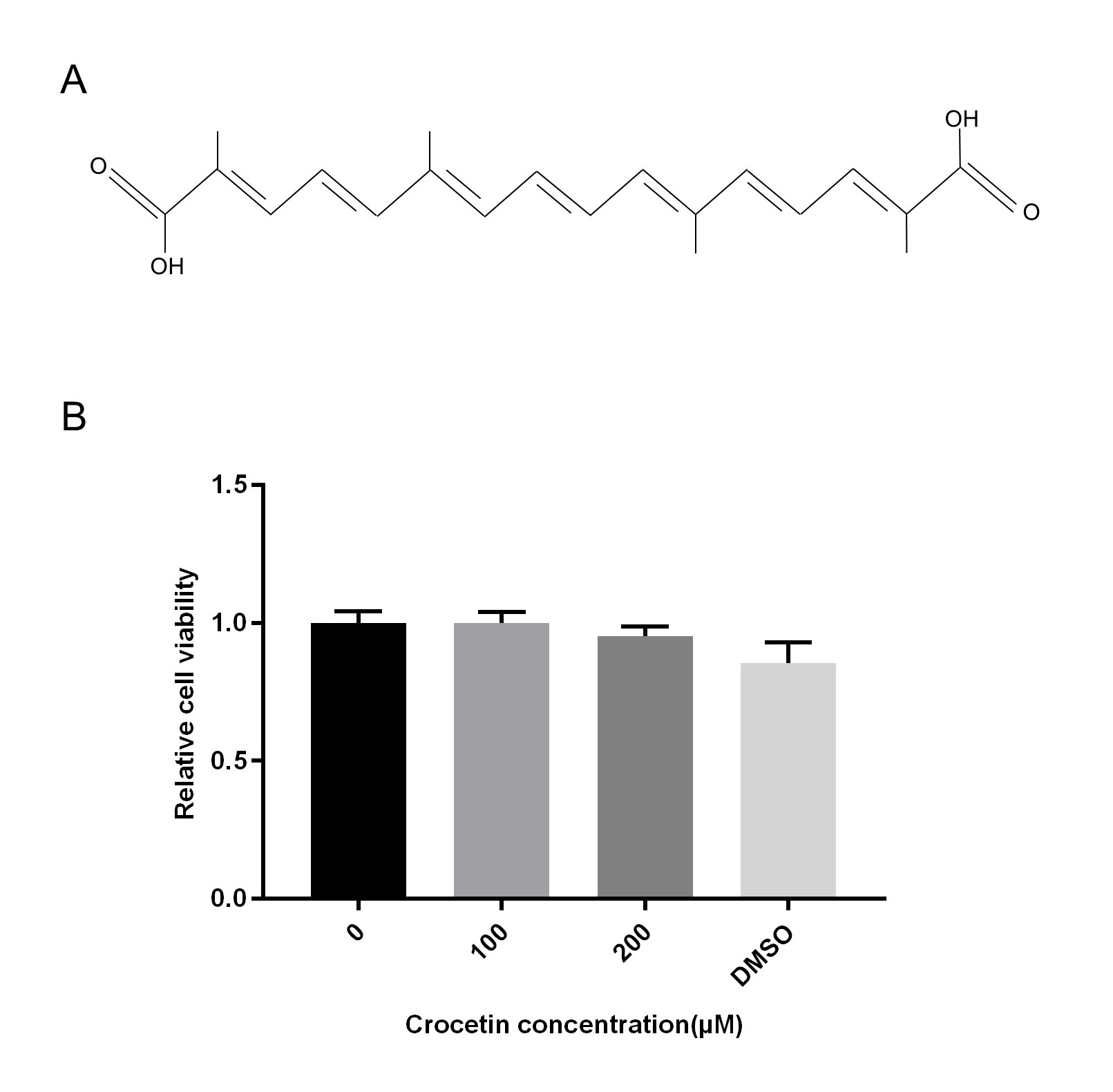

Supplement: Supplementary file 1 [file ijms-21-02949-s001.zip › supplementary files/Suupl. Figures/Supp2.jpg]

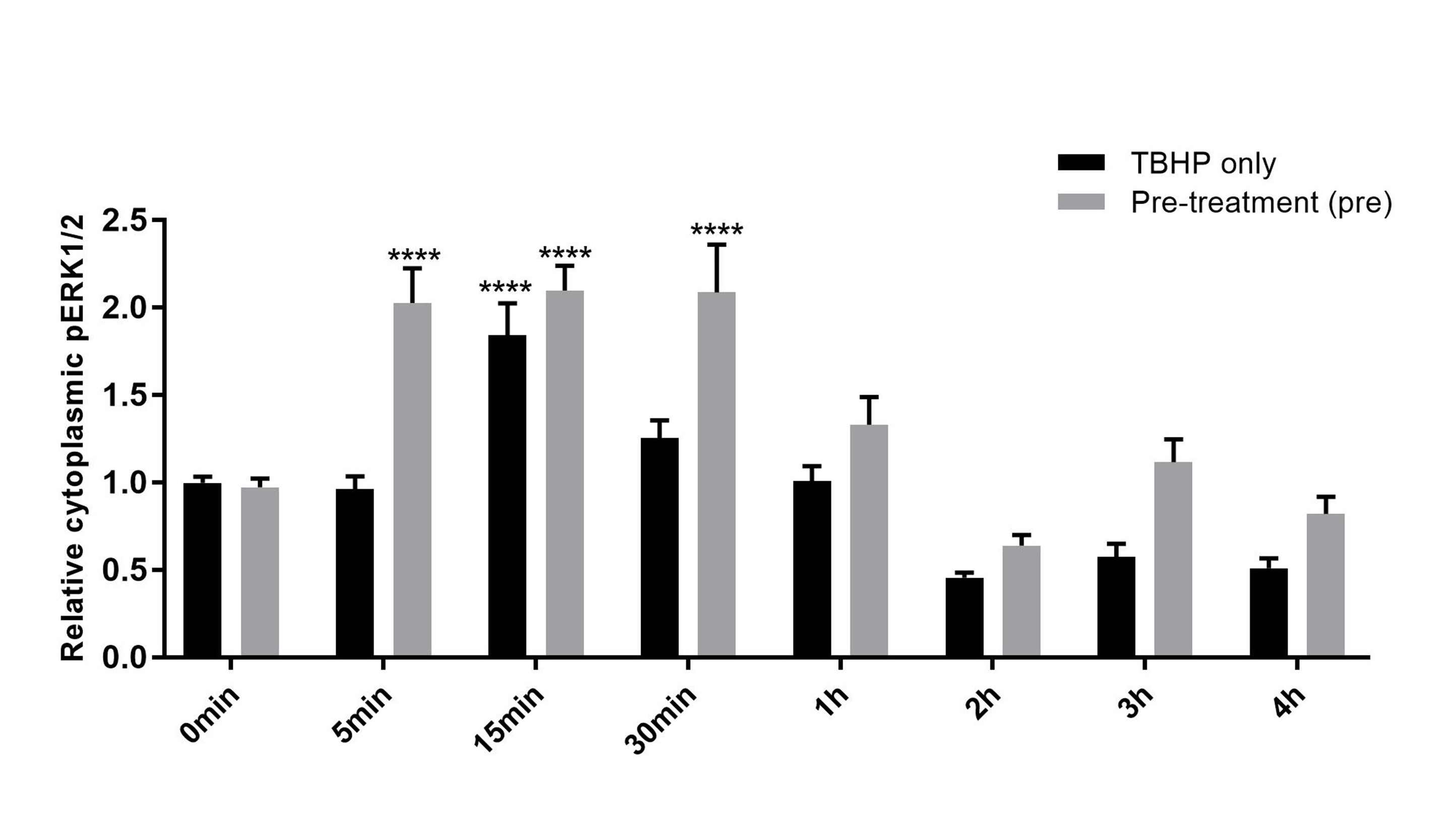

Supplement: Supplementary file 1 [file ijms-21-02949-s001.zip › supplementary files/Suupl. Figures/Supp3.jpg]

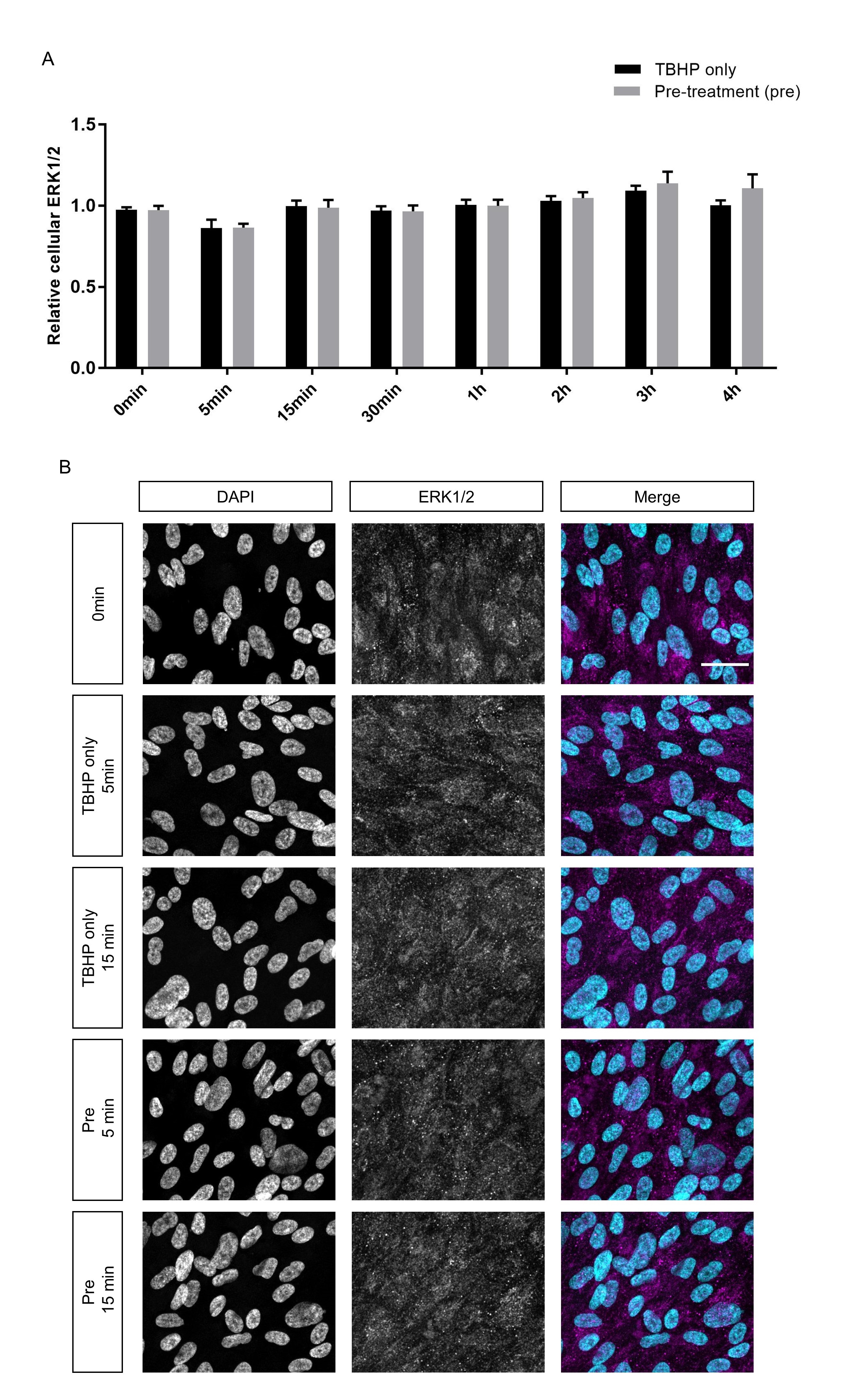

Supplement: Supplementary file 1 [file ijms-21-02949-s001.zip › supplementary files/Suupl. Figures/Supp4.jpg]

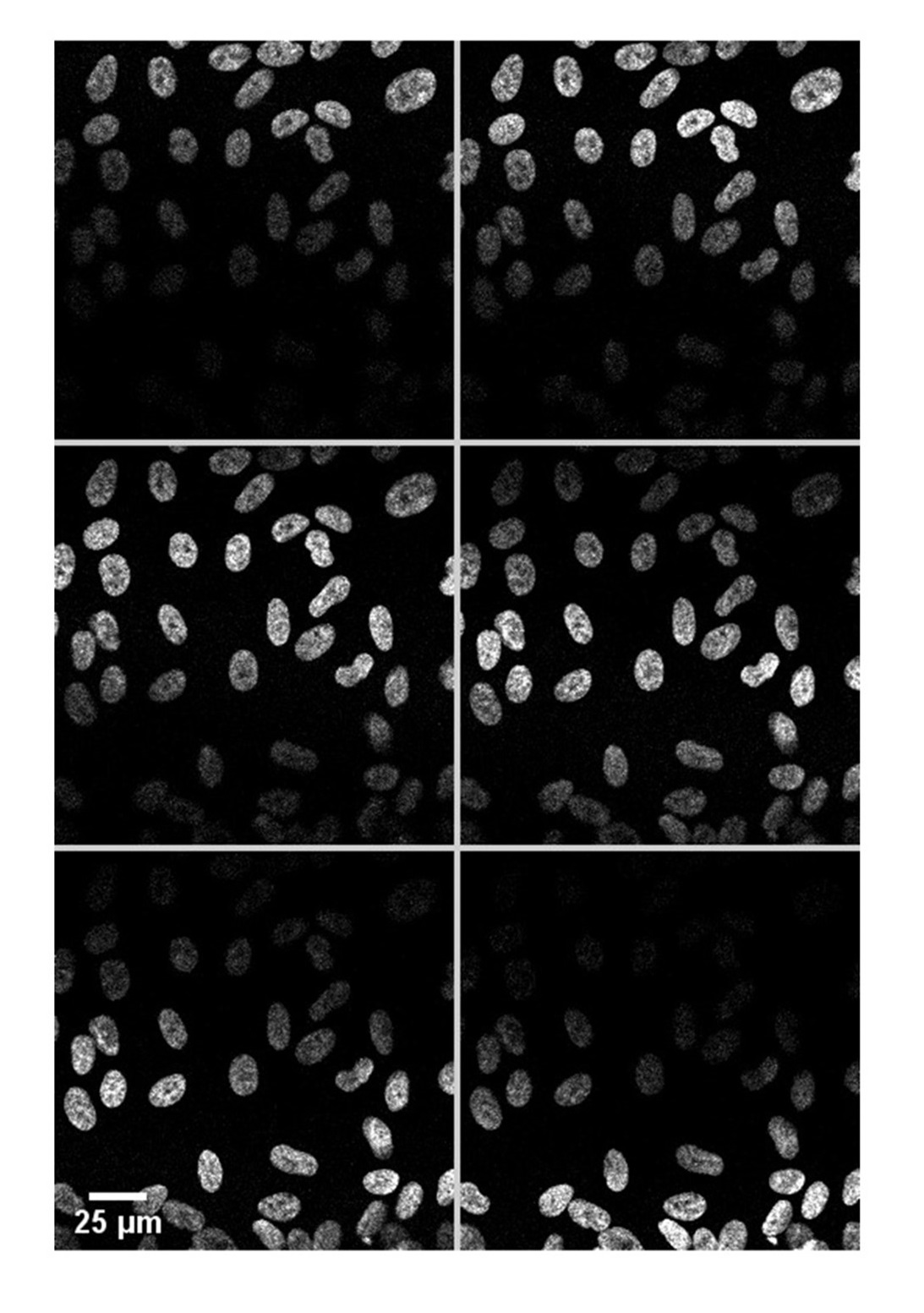

Supplement: Supplementary file 1 [file ijms-21-02949-s001.zip › supplementary files/Suupl. Figures/Supp5.jpg]

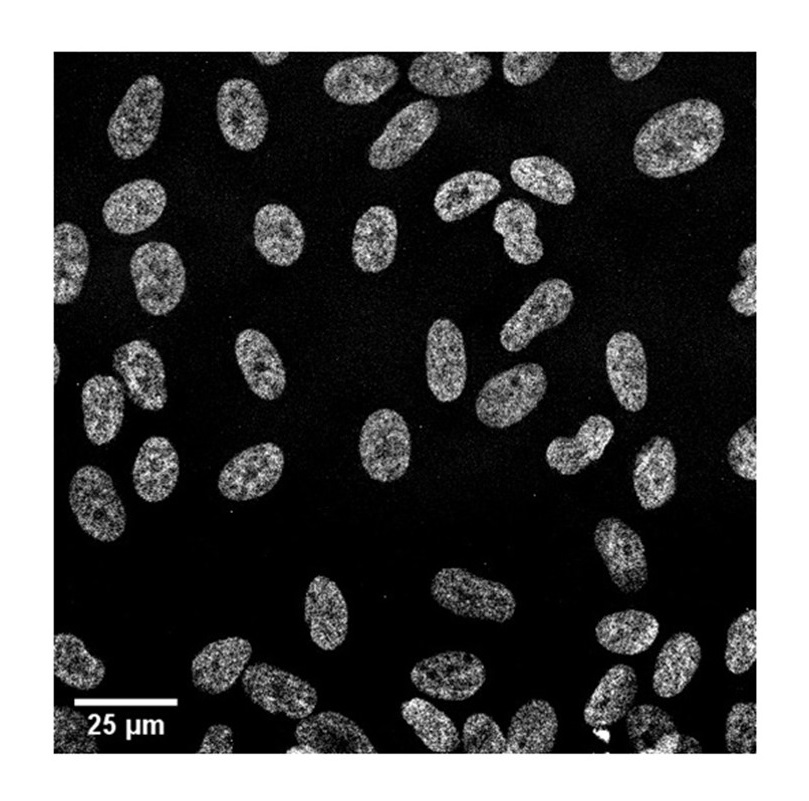

Supplement: Supplementary file 1 [file ijms-21-02949-s001.zip › supplementary files/Suupl. Figures/Supp6.jpg]
