# Supplementary material for: Crocetin Prevents RPE Cells from Oxidative Stress through Protection of Cellular Metabolic Function and Activation of ERK1/2
Source: Int J Mol Sci. 2020 Apr 22;21(8):2949. doi: 10.3390/ijms21082949 (PMC7215651; doi:10.3390/ijms21082949)
Supplement: Supplementary file 1 [file ijms-21-02949-s001.zip › supplementary files/Supplementary Material-revision-reviewers comments (1).docx]

**Supplementary Materials:**

**Supplementary figure 1.** Effect of TBHP on ARPE19 cell viability. Cell viability was determined by MTS assay 0 or 12h after exposure (4h) to different concentrations (0, 75, 150 and 300µM) of TBHP. 300µM TBHP significantly reduced cell viability 12h after end of exposure time. Data shown as mean ± SEM (n=3). P value <0.05 (*), <0.0001 (****) (One-way ANOVA, Dunnett’s multiple comparison test).

**Supplementary figure 2. crocetin structure and cytotoxicity.** Chemical structure of crocetin (A) (PubChem Public Database, http://pubchem.ncbi.nlm.nih.gov). ARPE19 cells were treated with crocetin (100 or 200µM) or 0.2% DMSO (solvent control) for 24h and cell viability was measured using MTS assay. No significant differences in cell viability was observed (n=4) (B) (One-way ANOVA, Dunnett’s multiple comparison test).

**Supplementary figure 3.** Effect of crocetin on cytoplasmic activation of ERK1/2 in ARPE19 cells. The relative amount of phosphorylated (p) ERK1/2 in the cytoplasm of TBHP exposed ARPE19 cells with crocetin pre-treatment was determined. While pERK1/2 was significantly increased in TBHP-only cells at 15 min in comparison to controls (0 min), crocetin-pre-treated cells showed an increase in pERK1/2 already at the 5 min interval that continued until 30 min, similar to the levels of nuclear pERK1/2 (see Fig. 5A). Data are shown as mean ± S.E.M. P value : <0.05 (*), <0.01 (**), <0.001 (***), <0.0001 (****) n=2, (One-way ANOVA, Dunnett’s multiple comparison test).

**Supplementary figure 4.** Effect of crocetin on ERK1/2 signal in ARPE19 cells. Comparison of ERK1/2 signal within ARPE19 cells showed no significant difference between TBHP-only and crocetin pre-treatment groups in any of the investigated time points (A), revealing that the expression levels of ERK1/2 did not alter and only their activation (as measured by phosphorylated (p) ERK1/2) was changed following TBHP and crocetin treatments. Immunocytochemistry results (B) of ARPE19 cells in control (0min), TBHP at 5min, TBHP at 15min, as well as TBHP plus crocetin pre-treatment at 5 and 15 min are shown for DAPI and total ERK1/2 (B; the third column represents merged images of DAPI and ERK1/2). ERK1/2 levels in TBHP-only and pre-treatment groups were not changed in comparison to controls (0min). Scale bar: 40µM.

**Supplementary Fig. 5.** Z-stack representation of DAPI staining. The curvature of the well bottom is far larger than the thickness of the optical sections. Therefore, the cells appear on different focal planes.

**Supplementary Fig. 6.** In-focus image using EDF. A complex wavelet transform is utilized to project the in-focus pixels in the slices on a 2D image to compensate the curvature of the bottom of the well.
